# Indoor radon exposure and health risks in a community proximal to gold mine tailings in Gauteng Province, South Africa: a cross-sectional study

**DOI:** 10.1007/s10653-025-02677-5

**Published:** 2025-08-07

**Authors:** Khathutshelo Vincent Mphaga, Wells Utembe, Thokozani P. Mbonane, Phoka C. Rathebe

**Affiliations:** 1 Department of Environmental Health, Faculty of Health Sciences, University of Johannesburg, Doornfontein Campus, Johannesburg, 2006 South Africa; 2 National Health Laboratory Service, Toxicology and Biochemistry Department, National Institute for Occupational Health, Johannesburg, 2000 South Africa

**Keywords:** Indoor radon exposure, Chronic obstructive pulmonary diseases (COPD), Leukemia, Gauteng, Lung cancer

## Abstract

Exposure to indoor radon presents a significant risk for lung cancer and is also suspected to be associated with other health issues such as chronic obstructive pulmonary diseases (COPD) and leukemia. This study examined the association between indoor radon exposure and self-reported cases of lung cancer, COPD, and leukemia among individuals living in close proximity to gold mine tailings, a known source of high radon levels. A cross-sectional study was carried out among residents living near or further away from gold mine tailings in Gauteng, South Africa. A total of 331 residents took part in an interviewer-administered questionnaire survey on socio-demographic characteristics, smoking habits, occupational exposures, adverse health problems, and other factors related to lung cancer, COPD, and leukemia. Subsequently, their homes were tested for indoor radon concentration on the lowest livable floor over a two-hour period from June to July 2023 using AlphaE radon monitors. Logistic regression was employed to calculate odds ratios (OR) for lung cancer, COPD, and leukemia based on indoor radon exposure, dwelling location, smoking habits, and occupational exposures. The study found significantly higher indoor radon levels in the exposed group (103.30 ± 94.91 Bq/m^3^) compared to the control group (65.19 ± 47.83 Bq/m^3^). The results indicated an association between lung cancer and residing near gold mine tailings, while indoor radon exposure was linked to leukemia. However, no association was found between indoor radon exposure and COPD. In regions impacted by gold mine tailings, it is crucial to implement efficient indoor radon mitigation measures and promote public consciousness about the health hazards linked to indoor radon exposure. There is a need to motivate affected residents to adopt proactive steps to lower indoor radon concentrations, including enhancing ventilation practices.

## Introduction

Radon is a radioactive gas and a significant natural source of ionizing radiation for humans (Bersimbaev & Bulgakova, 2017). Discovered by Ernest Rutherford in 1899, it exists primarily in three isotopes: radon-219, radon-220, and radon-222, with radon-222 being the most stable and common concentrations (Eidy et al., 2024; Leuchner et al., 1989; Terblanche & Murray, 1991). It has a half-life of 3.8 days and is produced from the decay of radium-226 and uranium-238. Due to its half-life and properties as a dense, odorless, and colorless gas, radon can accumulate in poorly ventilated areas, especially in homes, leading to potentially dangerous levels (Terblanche & Murray, 1991; ICRP, 1993; World Health Organization (WHO), 2009; International Atomic Energy Agency (IAEA) 2019). Radon gas is commonly found in the ground beneath dwellings, some building materials, ground water, outdoor air, and natural gas. It can seep into buildings through cracks and gaps in the foundations and walls, as well as around service pipes. While radon itself is not directly harmful, its decay products, polonium-218 and polonium-214, emit alpha particles that can damage DNA, thereby increasing the risk of health problems (World Health Organization (WHO), 2009; Eidy et al., 2024). Radon daughter products are also radioactive and easily attach themselves to whatever they encounter. Exposure occurs primarily through inhalation and to a lesser extent through ingestion of radon or radon-laden dust particles (Terblanche & Murray, 1991; World Health Organization (WHO), 2009; Eidy et al., 2024.

Radon gas has been identified as a significant health hazard due to its well-documented association with lung cancer (Bersimbaev & Bulgakova, 2017). Classified as a Group 1 carcinogen by the International Agency for Research on Cancer (IARC), World Health Organization (WHO) and US Environmental Protection Agency (EPA), radon exposure is the leading cause of lung cancer in non-smokers and a second contributor for smokers (World Health Organization (WHO), 2009; Ruano-Ravina et al., 2017). Radon short-lived radioactive radon daughters, when inhaled readily attach to lung tissue, emitting alpha particles that damage cells and increase cancer risk (Eidy et al., 2024; Terblanche & Murray, 1991). The World Health Organization (WHO) recommends an indoor radon reference level of 100 Bq/m^3^, with a maximum acceptable level of 300 Bq/m^3^ in cases where reaching the lower level is impractical (World Health Organization (WHO), 2009). Notably, even low to moderate concentrations pose a risk, with studies suggesting between 8 and 16% increase in lung cancer risk for every 100 Bq/m^3^ increase in radon exposure (Darby et al., 2005; International Atomic Energy Agency (IAEA); ; ; 2019; Krewski et al., 2006; Stanley et al., 2019). Additionally, it has been confirmed that residential radon exposure is a risk factor for lung cancer even at levels below those recommended by international organizations (Lorenzo-Gonzalez et al., 2020). Fernández-Navarro et al. (2012) and Moshupya et al. (2019) found a correlation between residing near mining facilities and an elevated risk of lung cancer mortality, further emphasizing the potential health hazards associated with elevated radon levels in such residential settings. Previous research has suggested that the detrimental effects of residential radon exposure extend beyond lung cancer. Studies have demonstrated an association between high residential radon levels and an increased incidence of chronic obstructive pulmonary disease (COPD) (Turner et al., 2012; Wang et al., 2022; Yitshak-Sade et al., 2019). Although the evidence for a link between radon exposure and leukemia risk is primarily derived from ecological studies, these studies has suggested a potential association, particularly for chronic lymphocytic leukemia (Oancea et al., 2017; Zlobina et al., 2022).

The implementation of public health measures to address radon exposure has been a common practice globally, involving national surveys and awareness campaigns (World Health Organization (WHO), 2009; International Atomic Energy Agency, 2017; Pantelić et al, 2019; International Atomic Energy Agency (IAEA) 2019; Bochicchio et al., 2022. Despite the presence of distinct indoor radon risk factors such as gold mine tailings in South Africa (Maheso et al., 2023; Moshupya et al., 2023; Terblanche & Murray, 1991). South Africa has yet to establish similar initiatives. These tailings, composed of waste materials from gold extraction, often contain high levels of uranium, a precursor to radon gas (Chanda-Kapata, 2020). While colder climates tend to lead to higher radon exposure due to increased indoor time (Bersimbaev & Bulgakova, 2017; Stanley et al., 2019). South Africa’s climate may provide some advantage through improved ventilation (Terblanche & Murray, 1991). However, this potential benefit is offset by the cold Highveld winters and the absence of central heating in many homes (Leuchner et al., 1989). The Witwatersrand area in Gauteng, with a population of over a million, is of concern due to its high density and proximity to gold mine tailings containing high uranium concentrations (Schonfeld et al., 2014; Winde et al., 2019; Zupunski et al., 2023). These gold mine tailings, are a result of over 80 gold mines which has operated in this region since the eighteenth century, pose a significant health risk to residents (Iyaloo et al., 2020; Utembe et al., 2015; Zupunski et al., 2023). It is estimated that there are 600,000 tons of uranium contained in these tailings across the Witwatersrand basin, presenting a significant environmental health concern that demands immediate attention (Liefferink, 2017; Schonfeld et al., 2014).

Previous occupational studies, particularly among miners, have consistently shown an association between elevated radon levels and an increased risk of lung cancer (Chen et al., 2023; Edwards et al., 2014; Lane et al., 2019), COPD (Chen et al., 2023; Conde-Sampayo et al., 2020), and leukemia (Řeřicha et al., 2006). Considering that much of the general population spends a significant amount of time indoors, assessing various exposure pathways to carcinogens like radon gas is increasingly crucial, especially since levels of certain toxic substances can be higher indoors than outdoors (Fishbein, 1992). In South Africa, despite international regulations prohibiting settlements near such sites, historical planning oversights and inadequate environmental controls have resulted in communities residing close to gold mine tailings (Bobbins, 2013; Khanyile, 2016; International Human Rights Clinic 2016; Benchmarks Foundation, 2016; Nkosi et al, 2015). The presence of elevated uranium in the tailings and surrounding soil, combined with windblown dust and reports of higher respiratory illness rates in nearby communities, necessitates further investigation (Iyaloo et al., 2020; Nkosi et al., 2015a, 2015b; The Bench Marks Foundation, 2016). Few small-scale studies have confirmed as association between elevated indoor radon exposure amongst dwellings proximal gold mine tailings (Kamunda et al., 2017; Moshupya et al., 2019). However, epidemiological studies on radon concentration levels and associated health risks are currently lacking in South Africa (Schonfeld et al., 2014). To address this knowledge gap and inform public health interventions, well-designed epidemiological studies are urgently needed. Thus, the aim of this study was to investigate an association between radon exposure and self-reported risks of lung cancer, COPD, and leukemia among residents living in close proximity to gold mine tailings in Gauteng Province. The findings from this research can be used to develop targeted strategies to protect these vulnerable communities.

## Materials and methods

### Study design and population

A cross-sectional epidemiological study was carried out to investigate the association between indoor radon exposure and self-reported lung cancer, COPD and leukemia risk amongst residential homes near gold mine tailings in Gauteng, South Africa. Gauteng is the smallest, most populous (with a population of around 15.8 million) and the main economic hub of the nine provinces of South Africa (Bobbins, 2013; Statistics South Africa, 2023). The name “Gauteng” comes from a Sotho word meaning “gold,” reflecting the area’s history of gold mining. It is home to the Witwatersrand Basin, the largest gold mining area in the world (International Human Rights Clinic, 2016). The majority of urban and township areas in Gauteng have been established around the minefields (including mine dumps), creating a distinctive landscape different from other mining contexts abroad (Bobbins, 2013). In this study, Riverlea was identified as the exposed group due to its close proximity (0.1–2 km) to three dormant and active gold mine tailings storage facilities (Nwaila, 2020). The closest gold mine tailings storage facilities are located less than 200 m (m) away from the community (The Bench Marks Foundation , 2016; Kootbodien et al., 2019). Communities adjacent to gold mine tailings storage facilities are subjected to elevated dust fallout due to the dispersion of fine particles from these facilities (International Human Rights Clinic, 2016; Andraos et al., 2018; Kootbodien et al., 2019; Nkosi et al., 2021). Arid conditions have hindered the rehabilitation of partially vegetated tailings, while current mine dump reclamation for gold extraction has exacerbated dust pollution and deteriorated air quality (Kootbodien et al., 2019; The Bench Marks Foundation, 2016). Radon gas, potentially attached to dust particles released from these facilities, can be dispersed by wind and atmospheric turbulence, posing a risk to nearby homes. Additionally, heavy rains through runoffs have been reported to carry contaminants from the gold mine tailings, potentially including radon gas, from the mine tailings to nearby communities (International Human Rights Clinic, 2016). Riverlea is home to 16,226 residents, residing in 4,208 dwellings built around or near gold mine tailings from the past (The Bench Marks Foundation, 2016; Kootbodien et al., 2019). Riverlea was selected as an exposed area due to its proximity to gold mine tailings, which could potentially result in higher indoor radon exposure. This increased exposure may consequently lead to a higher risk of radon-induced lung cancer, COPD, and leukemia. The community of Orlando East was chosen as the unexposed (control/reference) group due to the absence of gold mines or a history of mining in the area, which minimizes the potential for radon exposure. The closest gold mine tailings are located more than 2 km away from Orlando East (Chiyangwa, 2020). Orlando East has a population of 68,210 people and 22,416 households (Frith, 2022). The Riverlea and Orlando East areas, located within the Witwatersrand Basin, present a predominantly flat topology characterized by expansive grassland ecosystems. Both regions experience a Cwb subtropical highland climate, representative of the Johannesburg locality, with mean annual temperatures fluctuating between 19 and 20 °C. The summer months are marked by moderate temperatures, averaging around 24 °C, while winter temperatures exhibit a decline, averaging approximately 13 °C. Precipitation in this area is around 750 mm annually, as referenced in Kamunda et al. (2017).

### Sample size and sampling procedure

**Fig. 1:**
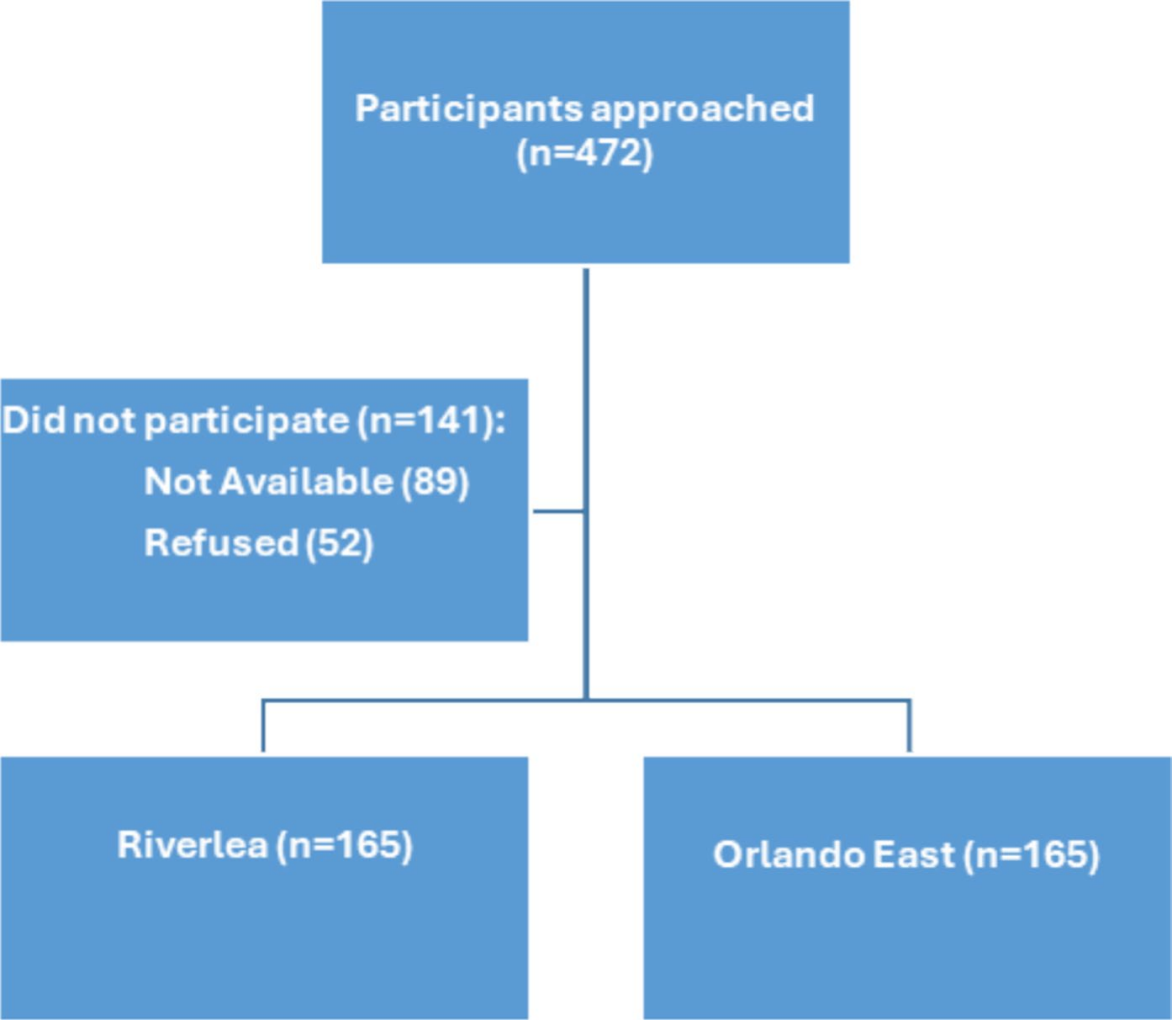
Participants distribution

The study included 7477 residential dwellings in Riverlea and Orlando East sourced from the City of Johannesburg Online Maps. Only residents from these two communities were included (City of Johannesburg, 2017). Random sampling was conducted using Microsoft Excel 365. The study involved 476 participants, with 238 individuals in both the exposed and control groups. The sample size was determined using EpiInfo Version 7 software, considering a population size of 7477 residential dwellings, an expected frequency of 20%, a 95% confidence level, and a 5% margin of error. The study permitted one resident per household, over the age of 18 and residing in the area for at least 5 years, to participate with informed consent. Ethical approval was obtained from the University of Johannesburg’s Faculty of Health Sciences Research Ethics Committee and the Higher Degree Committee. Out of 472 potential participants, 89 (18.9%) were unavailable, and 52 (11.0%) declined. A total of 331 individuals participated, resulting in a 70.1% participation rate, evenly distributed between Riverlea and Orlando East (See Fig. 1).

### Data collection tools and procedures

The researcher, along with two trained assistants who were familiar with the community and spoken language, collected data using structured interviewer-administered questionnaires and four AlphaE radon monitors. The data collection took place during the winter season, specifically from June to July 2023. Upon identifying a selected dwelling, we used a knock-on-the-door approach to target occupants who met the inclusion criteria. Data was gathered through in-person interviews with eligible participants residing in selected residences. Prior to data collection, informed consent was obtained. The interviews were conducted in English, with translation to local language provided when necessary. The structured questionnaire covered socio-demographic characteristics, housing conditions, occupational and smoking histories, and health status. The questionnaire development involved a comprehensive review of published literature, including sources such as the World Health Organization (World Health Organization (WHO), 2009) and the International Atomic Energy Agency (IAEA) 2019). Pilot testing was conducted with 16 participants from the study area to ensure the questionnaire’s clarity and suitability for the intended purpose. To guarantee reliability, the results from the pilot study were analyzed using Cronbach’s alpha, which yielded a coefficient of *α* > .7, indicating good internal consistency.

Indoor radon levels were assessed using AlphaE radon monitors deployed in participants’ homes. A single monitor was placed in a room where a participant spend most of his or her time, either the bedroom or living room, to evaluate indoor radon concentration. A single, two-hour measurement was conducted per residence in both control and exposed communities, with the resulting radon concentration expressed in becquerels per cubic meter (Bq/m^3^) recorded for analysis. This approach enabled efficient large-scale screening and helped to identify homes with potentially elevated radon levels that may require further investigation. This short-term measurement approach, supported by previous radon studies (Kamunda et al., 2017; Mirbag & Poursani, 2018), allows for effective preliminary assessments and acute exposure evaluations. The two-hour duration captures essential radon concentration data while minimizing disruption to participants and simplifying logistics for larger studies. AlphaE monitors were calibrated both by the manufacturer (Bertin Technologies) and a local laboratory (Selectech) to ensure accuracy. Calibration involved exposing the monitors to a known radon concentration in a sealed chamber alongside a reference unit (AlphaGUARD). The manufacturer and local calibration laboratory reported a precision of ± 10% at the 95% confidence level. All measurements were conducted according to the manufacturer’s protocols. AlphaE radon monitor placement followed manufacturer manual, US-EPA (1993), WHO (2009) and International Atomic Energy Agency (IAEA) (2019). Protocols. AlphaE radon mionitor was placed at least 1.5 m above the floor, more than 0.5 m from windows and ceilings, and 0.4 m from walls, while avoiding airflow disruptions from heating, ventilation, and fluctuating door and window use (Kamunda et al., 2017; Mirbag & Poursani, 2018). Data collected was then transferred to a PC for analysis using DataVIEW software.

### Statistical analysis

We conducted a bivariate descriptive analysis to examine the distribution of the study variables based on the exposed and control group. Subsequently, we utilized univariate and multivariate logistic regression with radon-induced health problems (self-reported lung cancer, COPD, and leukemia) as the dependent variable and residential radon exposure and dwelling location (Riverlea and Orlando East) as the independent variables. Initially, indoor radon exposure was analyzed as a continuous variable and then as a categorical variable with two categories (≤ 100 Bq/m^3^ and ≥ 100 Bq/m^3^). We explored effect modification between indoor radon exposure and other influencing factors (sex, age, tobacco smoking, type of fuel used at home, and occupational history) on the self-reported health problems by including a multiplicative term in the model. Logistic regression results were presented as crude and adjusted odds ratios with a 95% confidence interval. For bivariate analysis, independent variables with a *p*-value of < 0.20 were considered significant and included in the multivariate analysis. In the multivariate analysis, a *p*-value of < 0.05 was considered significant. Additional independent variables were included in the multivariate analysis based on established facts from literature or known associations from previous studies. All analyses were conducted using STATA 18 (Stata Corp., College Station, TX, USA).

## Results

### Demographic characteristics of the participants

Out of 476 potential subjects identified for the study, 331 participated in an interviewer-administered questionnaire survey, and valid indoor radon measurements were obtained from their respective dwellings. Ultimately, data from both radon measurements and questionnaire surveys were available for 166 exposed subjects (102 females and 64 males) and 165 control subjects (98 females and 67 males), following a 1:1 matching design (Table 1). The majority of the participants were female, comprising 102 (61.4%) exposed individuals and 98 (59.4%) controls. The age distribution differed notably between the two groups. The exposed group had a higher proportion of younger residents, with 24 (14.5%) participants under the age of 39 years. In contrast, the control group had a significantly higher percentage of older residents, with 100 (60.6%) participants who were 60 years and above. Educational attainment was similar between the two groups, with most participants having completed high school. However, the control group had a slightly higher proportion of individuals with tertiary education, 27 (16.4%) compared to 13 (7.8%) in the exposed group. Employment rates were comparable, but the control group had a higher unemployment rate (84.1% compared to 76.5% in the exposed group). Both communities primarily resided in brick houses. Notably, a significantly higher percentage of the control group (89.7%) had lived in their current homes for over 31 years compared to the exposed group (70.5%). Ventilation practices were comparable between the two groups, with frequent ventilation being the norm. Radon testing was uncommon in both communities, with less than 2% of participants reporting prior testing. For detailed information regarding the dwelling characteristics of the participants, please refer to our previous publication (Mphaga et al., 2024).

**Table 1:** Demographic characteristics of the participants

		Riverlea n = 166 (50.2%)	Orlando east n = 165 (49.8%)
Sex	Female	102 (61.4%)	98 (59.4%)
	Male	64 (38.6%)	67 (40.6%)
Age	Below 39 Years	24 (14.5%)	13 (7.9%)
	40–49 Years	33 (19.9%)	24 (14.5%)
	50–59 Years	45 (27.1%)	28 (17.0%)
	60 Years and above	64 (38.5%)	100 (60.6%)
Marital status	Single	59 (35.5%)	81 (49.1%)
	Married	65 (39.2%)	70 (42.4%)
	Divorced	12 (7.2%)	1 (0.6%)
	Separated	1 (0.6%)	3 (1.8%)
	Windowed	29 (17.5%)	10 (6.1%)
	Black African	5 (3.0%)	163 (98.8%)
Race	Colored	159 (95.8%)	1 (0.6%)
	Indian/Asian	2 (1.3%)	0 (0.0%)
	White	0 (0.0%)	1 (0.6%)
	Less than high school	59 (35.5%)	40 (24.2%)
Education	High school completed	94 (56.6%)	98 (59.4%)
	Tertiary completed	13 (7.8%)	27 (16.4%)
Employment	Yes	39 (23.5%)	26 (15.9%)
	No	127 (76.5%)	138 (84.1%)
	Less than 10 years	15 (9.0%)	7 (4.2%)
Years in the same house	11–20 years	13 (7.8%)	2 (1.2%)
	21–30 years	21 (12.7%)	8 (4.9%)
	More than 31 years	117 (70.5%)	148 (89.7%)
House type	Brick house	166 (100.0%)	159 (96.4%)
	Other (i.e. shack)	0 (0.0%)	6 (3.6%)
	Often	149 (89.8%)	107 (64.8%)
Ventilation	Sometimes	15 (9.0%)	30 (18.2%)
	Rarely	2 (1.2%)	28 (17.0%)
	Less than 10 h	34 (20.4%)	12 (7.3%)
Hours inside the house per day	11–20 h	45 (27.2%)	96 (58.2%)
	More than 21 h	87 (52.4%)	57 (34.5%)
Tested for radon in the past	Yes	3 (1.8%)	3 (1.8%)
	No	163 (98.2%)	164 (98.2%)

### Risk factors

The following section (Table 2) compared the exposure factors and smoking habits of the populations in the exposed and control groups. The main aim was to identify variations in exposure risks and smoking behaviors between these two groups. While there were a limited number of participants reporting exposure to carcinogens, irritants, and toxins in both groups, a significant disparity was observed between the exposed and control groups. The analysis revealed a significantly higher prevalence of occupational exposure to these substances among the exposed individuals, with 21 individuals (n = 21; 12.7%) compared to only 3 individuals (n = 3; 1.8%) in the control group. A small number of participants from both communities reported working in underground mines, indicating that underground mining was not a significant occupational exposure factor for either population. The study also evaluated smoking habits across various dimensions, including current smoking status, household smoking, and indoor tobacco smoke exposure. The findings indicated a substantially higher percentage of current smokers in the exposed group (n = 97; 58.4%) compared to the control group (n = 44; 26.7%), suggesting a higher prevalence of smoking in the exposed group. Furthermore, a higher proportion of households in the exposed group had at least one smoker (n = 105; 63.3%) compared to the control group (n = 80; 49.1%). Moreover, a greater percentage of exposed households (n = 76; 46.3%) were exposed to indoor tobacco smoke compared to the control group (n = 48; 29.3%). Among smokers, a higher proportion of exposed residents reported a longer duration of smoking, with a significant number having smoked for over 33 years (n = 26; 15.7%) compared to only 1 individual (0.6%) in the control group, indicating a more established smoking habit in this community.

**Table 2:** Risk factors

		Riverlea n = 166 (50.2%)	Orlando east n = 165 (49.8%)
Occupational exposure to carcinogens, irritants, and toxins	Yes	21 (12.7%)	3 (1.8%)
	No	145 (87.3%)	162 (98.2%)
Years of exposure	Less than 20 years	17 (10.3)	2 (1.2%)
	More than 30 years	4 (2.4%)	1 (0.6%)
	Not applicable	145 (87.3%)	162 (98,2%)
	Yes	2 (1.2%)	`2 (1.2%)
Have you worked in underground mine	No	164 (98.8%)	162 (98.8%)
	Never smoker	57 (34.4%)	115 (69.7%)
Tobacco smoking	Ex-smoker	12 (7.2%)	6 (3.6)
	Current smoker	97 (58.4%)	44 (26.7%)
Does anyone in the household smoke?	Yes	105 (63.3%)	80 (49.1%)
	No	61 (36.7%)	83 (50.9%)
	One	43 (25.9%)	43 (26.0%)
Number of current smokers	More than 2	71 (42.8%)	42 (25.5%)
	None	52 (31,3%)	80 (48.5%)
Tobacco smoking indoors	Yes	76 (46.3%)	48 (29.3%)
	No	88 (53.7%)	116 (70.7%)
	None	57 (34.3%)	116 (70.3%)
Number of cigarettes per day	≤ 10 Cigarette/day	85 (51.2%)	37 (22.4%)
	11- 21 Cigarette/day	24 (14.5%)	11 (6.7%)
	≥ 22 Cigarettes/day	0 (0.0%)	1 (0.6%)
	None	56 (33.7%)	115 (69.7%)
	Less than 10 years	38 (22.9%)	18 (10.9%)
Years of smoking	11–21 years	30 (18.0%)	21 (12.7%)
	22–32 years	16 (9.6%)	7 (4.3%)
	More than 33 years	26 (15.7%)	4 (2.4%)

### Radon measurements

Indoor radon levels were compared between exposed and control group. Radon measurements were obtained from the dwellings in the exposed and control group (Table 3). The radon levels in both groups exhibited a wide range, as evidenced by the minimum and maximum values. The exposed group had a significantly higher mean radon level (103.30 ± 94.91 Bq/m^3^) compared to the control group (65.19 ± 47.83 Bq/m^3^). When considering the geometric mean, which is less influenced by outliers, the disparity between the groups was less pronounced. The exposed group had a geometric mean of 81.59 Bq/m^3^, while the control group had a geometric mean of 52.49 Bq/m^3^. A substantial proportion of dwellings in both groups exceeded the World Health Organization (WHO) reference level of 100 Bq/m^3^. In the exposed group, (n = 68; 41%) of the dwellings exceeded this threshold, whereas in the control group, (n = 31; 19%) exceeded it. Indoor radon levels were significantly linked to the location of the homes, with homes near (≤ 2 km) gold mine tailings (exposed) experiencing elevated indoor radon levels compared to those farther away (≥ 2 km) from the mine (control). A comprehensive analysis of indoor radon determinants, including construction materials, the age of dwellings, and other relevant factors, has been extensively discussed in our related publication (Mphaga et al., 2024).

**Table 3:** Residential radon gas concentration for exposed and control group

	Exposed	Control
Minimum	11.07	0
Maximum	1078.85	379.13
Arithmetic mean	103.30	65.19
Standard deviation	94.91	47.83
Geometric mean	81.59	52.49
Dwellings exceeding WHO Reference level of 100 Bq/m^3^	68 (68, 7%)	31 (31, 3%)

### Adverse health problems

This study compared the frequency of various respiratory health conditions between a group exposed to high radon levels and a control group (Table 4). Despite most participants in both groups having access to healthcare, a significantly higher percentage of individuals in the control group (n = 80; 50.6%) reported easy access compared to the exposed group (n = 57; 26.6%). The exposed group had significantly higher rates of several respiratory diseases, including tuberculosis (7.2% vs. 8.5%), bronchitis (8.4% vs. 4.2%), and asthma (9.6% vs. 3.7%). Additionally, a significant proportion of the exposed group reported persistent cough, shortness of breath, and unexplained weight loss compared to the control group. The control group reported no cases of COPD, while a single case was identified in the exposed group. The prevalence of leukemia was low in both groups, with no significant differences observed. The exposed group had a significantly higher prevalence of lung cancer diagnoses (n = 3; 1.8%), compared to the control group (n = 1, 0.6%). Moreover, a notably higher percentage of the exposed group had relatives diagnosed with lung cancer (n = 27; 16.3%) compared to the control group (n = 2; 1.2%). The exposed group also had a significantly higher rate of deaths due to lung cancer among relatives (n = 26; 15.7%) compared to the control group (n = 2; 1.2%). The majority of participants in both groups expressed a willingness to participate in lung cancer screening, with slightly higher rates in the exposed group (n = 153; 92.7%) compared to the control group (n = 143; 87.2%).

**Table 4:** Adverse health problems for exposed and control group

		Riverlea n = 166 (50.2%)	Orlando east n = 165 (49.8%)
Access to health care	Difficult	70 (42,4%)	44 (27.9%)
	Moderate	38 (23.0%)	34 (21.5%)
	Easy	57 (26.6%)	80 (50.6%)
Have you ever been diagnosed with TB	Yes	12 (7.2%)	14 (8.5%)
	No	154 (92.8%)	151 (91.5%)
Have you ever been diagnosed with bronchitis	Yes	14 (8.4%)	7 (4.2%)
	No	152 (91.6%)	158 (95.8%)
Have you ever been diagnosed with asthma	Yes	16 (9.6%)	6 (3.7%)
	No	150 (90.4%)	158 (96.3%)
Relative diagnosed with asthma	Yes	38 (22.9%)	22 (13.3%)
	No	128 (77.1%)	143 (86.7%)
Have you ever been diagnosed with COPD	Yes	1 (0.6%)	0 (0.0%)
	No	165 (99.4%)	165 (100.0%)
Have you ever been diagnosed with leukemia	Yes	2 (1.2%)	1 (0.6%)
	No	164 (98.8%)	164 (99.4%)
Relative with leukemia	Yes	1 (0.6%)	0 (0.0%)
	No	165 (99.4%)	165 (100.0%)
Have you been diagnosed with lung cancer	Yes	3 (1.8%)	1 (0.6%)
	No	163 (98.2%	164 (99.4%)
Relative diagnosed with lung cancer	Yes	27 (16.3%)	2 (1.2%)
	No	139 (83.7%)	163 (98.8%)
Have you lost a relative due to lung cancer	Yes	26 (15.7%)	2 (1.2%)
	No	140 (84.3%)	163 (98.8%)
Do you experience persistent cough	Yes	48 (28.9%)	7 (4.2%)
	No	118 (71.1%)	158 (95.8%)
Do you experience shortness of breath	Yes	37 (22.3%)	7 (4.2%)
	No	129 (77.7%)	158 (95.8%)
Do you cough blood	Yes	2 (1.2%)	0 (0.0%)
	No	164 (98.8%)	165 (100.0%)
Unexplained weight loss	Yes	4 (2.4%)	0 (0.0%)
	No	162 (97.6%)	165 (100.0%)
Repeated respiratory infections	Yes	15 (9.0%)_	0 (0.0%)
	No	151 (91.0%)	165 (100.0%)
Willingness to participate in lung cancer screening	Yes	153 (92.7%)	143 (87.2%)
	No	12 (7.3%)	21 (12.8%)

### Association between various risk factors and lung cancer

Logistic regression analysis was employed to calculate crude and adjusted odds ratios (ORs) for lung cancer risk, with 95% confidence intervals (CIs) (Table 5). The results revealed a significant association between dwelling location and lung cancer risk (crude OR = 0.063, 95% CI = 0.015–0.270, *p* < 0.001; adjusted OR = 0.048, 95% CI = 0.010–0.210, *p* < 0.001). However, no statistically significant association was observed between indoor radon exposure and lung cancer risk, neither in the crude analysis (OR = 1.000, 95% CI = 0.995–1.004, *p* = 0.983) nor in the adjusted analysis (OR = 0.995, 95% CI = 0.988–1.003, *p* = 0.250). Active smoking and passive smoking were also considered as potential risk factors. While the crude analysis suggested a non-significant association with lung cancer risk for both active (OR = 1.365, 95% CI = 0.917–2.032, *p* = 0.125) and passive smoking (OR = 1.604, 95% CI = 0.746–3.447, *p* = 0.226), the adjusted analyses revealed that these associations were attenuated and no longer statistically significant (active smoking: OR = 0.980, 95% CI = 0.587–1.636, *p* = 0.939; passive smoking: OR = 1.104, 95% CI = 0.419–2.908, *p* = 0.841). Occupational exposure to carcinogens, irritants, and toxins emerged as a significant risk factor for lung cancer in the adjusted analysis (OR = 7.545, 95% CI = 1.799–29.013, *p *= 0.031). This finding suggests that occupational exposure may play a more substantial role in lung cancer risk.

**Table 5:** Estimated odds ratios for lung cancer in relation to various factors

Risk factors	Crude odds ratio (95% CI)	*P*-value	Adjusted odds ratio (95% CI)	*P*-value
Dwelling location	0.063 (0.015–0.270)	≤ 0.001*	0.0477 (0.010–0.210)	≤ 0.001*
Indoor radon exposure	1.000 (0.995–1.004)	0.983	0.995 (0.988–1.003)	0.250
Active smoking	1.365 (0.917–2.032	0.125	0.980 (0.587–1.636)	0.939
Passive smoking	1.604 (0.746–3.447)	0.226	1.104 (0.419–2.908)	0.841
Occupational exposure	0.104 (0.071–0.153)	≤ 0.001*	7.545 (1.799–2.013)	0.031

### Association between various risk factors and leukemia

To assess the association between various risk factors and leukemia incidence, a comparative analysis was conducted between the exposed and control groups. The results, presented in Table 6, highlight significant disparities in exposure to several risk factors. Individuals residing in the exposed community exhibited a significantly higher risk of leukemia compared to the control group, as indicated by a crude odds ratio of 0.178 (95% CI: 0.072–0.442, *p* ≤ 0.001). However, after adjusting for confounding factors, this association was no longer statistically significant, suggesting that the initial association might be attributable to other factors. A consistent, strong association was observed between indoor radon exposure and the risk of leukemia. Both the crude and adjusted odds ratios were significantly elevated, indicating a 6.7% and 7.1% increased risk of leukemia for each 100 Bq/m^3^ increase in radon exposure, respectively. While active smoking did not exhibit a significant association with leukemia risk in the adjusted analysis, passive smoking was marginally associated with an increased risk. The crude odds ratio for passive smoking was 1.531 (95% CI: 0.746–3.447, *p* = 0.242), but after adjusting for confounders, the association weakened to 1.293 (95% CI: 0.886–1.601, *p* = 0.068). The analysis revealed a non-significant association between occupational exposure and leukemia risk. The crude odds ratio was 1.438 (95% CI: 0.781–2.645, *p* = 0.243), and the adjusted odds ratio remained non-significant at 1.694 (95% CI: 0.591–4.858, *p *= 0.327).

**Table 6:** Estimated odds ratios for leukemia in relation to various factors

Risk factors	Crude odds ratio (95% CI)	*P*-value	Adjusted odds ratio (95% CI)	*P*-value
Dwelling location	0.178 (0.072–0.442)	≤ 0.001*	0.342 (0.075–1.549)	0.164
Indoor radon exposure	1.067 (1.044–1.089)	≤ 0.001*	1.071 (1.045–1.097)	≤ 0.001*
Active smoking	1.237 (0.862–1.777	0.249	0.977 (0.608–1.570	0.171
Passive smoking	1.531 (.746–3.447)	0.242	1.293 (0.886–1.601)	0.068
Occupational exposure	1.438 (0.781–0.645)	0.243	1.694 (0.591–4.858)	0.327

### Association between various risk factors and COPD

The present study examined the association between several risk factors and COPD. To assess these relationships, a comparative analysis was conducted between exposed and control groups (Table 7). Dwelling Location: While initial analysis suggested a potential association between dwelling location and COPD risk (crude odds ratio: 2.440, *p*-value: 0.133), this association was not sustained after adjusting for confounding factors (adjusted odds ratio: 1.022, *p*-value: 0.581). Neither the crude nor adjusted analyses revealed a statistically significant association between indoor radon exposure and COPD risk. Both active and passive smoking were investigated as potential risk factors. Active smoking showed a non-significant crude association (crude odds ratio: 1.601, *p*-value: 0.458) but a slightly attenuated adjusted association (adjusted odds ratio: 0.842, *p*-value: 0.827). Passive smoking, on the other hand, exhibited a non-significant crude association (crude odds ratio: 3.327, *p*-value: 0.328) and a marginally significant adjusted association (adjusted odds ratio: 2.652, *p*-value: 0.520). The most notable finding was a significant association between occupational exposure and COPD risk. While the crude analysis indicated a non-significant association (crude odds ratio: 1.243, *p*-value: 0.311), the adjusted analysis revealed a significantly increased risk of COPD among individuals exposed to occupational hazards (adjusted odds ratio: 2.201–2.662, *p*-value: 0.214).

**Table 7:** Association between various risk factors and COPD

Risk factors	Crude odds ratio (95% CI)	*P*-value	Adjusted odds ratio (95% CI)	*P*-value
Dwelling location	2.440 (0.133–0.396)	≤ 0.001*	1.022 (0.988–1.057)	0.581
Indoor radon exposure	1.003 (0.996–1.001)	0.404	1.001 (0.994–1.008)	0.758
Active smoking	1.601 (0.463–5.541)	0.458	0.842 (0.179–3.955)	0.827
Passive smoking	3.327 (0.299–3.087)	0.328	2.652 (0.135–1.963)	0.520
Occupational exposure	1.243 (0.146–0.788)	0.311	0.001 (2.201–2.662)	0.214

## Discussion

### Indoor radon exposure

The current study revealed a substantial disparity in radon exposure between the exposed and control groups. The exposed group exhibited significantly higher mean radon levels compared to the control group. Nonetheless, a substantial proportion of dwellings in both groups exceeded the WHO reference level of 100 Bq/m^3^. The observed levels in the exposed group were comparable to those reported in previous studies conducted within residential dwellings (Kamunda et al., 2017; Moshupya et al., 2019; Wang et al., 2022). Moreover, the elevated indoor concentrations in the exposed group were comparable to those observed in occupational studies (Martin-Gisbert et al., 2023). This suggests that individuals residing proximal gold mine tailings may be exposed to radon levels that are comparable to those encountered in occupational settings, potentially increasing their risk of health adverse effects. Several studies have reported that a significant percentage of residential dwellings, particularly in regions with high uranium content in the soil, such as proximal gold mine tailings exceed recommended radon levels (Mlay & Makundi, 2018; Mohammed, 2018; Yazzie et al., 2020). These findings underscore the importance of radon mitigation strategies to protect public health.

### Lung cancer

In this cross-sectional analysis, we utilized logistic regression to explore the correlation between indoor radon exposure and the incidence of lung cancer, while also accounting for variables such as dwelling location, tobacco use, and occupational exposures. Despite significantly elevated indoor radon levels in the exposed cohort, our study did not reveal a statistically significant association between radon exposure and lung cancer risk, neither in unadjusted nor adjusted models. This outcome is particularly intriguing given the well-documented causal relationship established in extensive epidemiological literature (Auvinen et al., 1996; Barros-Dios et al., 2002; Bochicchio et al., 2000; Gariazzo et al., 2021; Ha et al., 2017; Hassfjell et al., 2017; Kim et al., 2018; Lee et al., 2015; Torres-Durán et al., 2014). Interestingly, positive correlations between radon exposure and lung cancer have been reported in studies conducted in residential contexts near gold mining areas in South Africa and Cameroon (Moshupya et al., 2023; Nkoulou et al., 2023). The divergence between our findings and the broader consensus, which includes positive associations from previous studies, can be ascribed to various methodological and contextual factors. Large-scale case–control studies and pooled radon investigations, such as those by Krewski et al. (2006) and Darby et al. (2005), leverage extensive sample sizes, comprehensive data drawn from national radon surveys, and robust mortality data, thereby enhancing statistical power (Keith et al., 2012). For instance, Krewski et al. (2006) quantified a lung cancer risk increment of 11% for each 100 Bq/m^3^ increase in radon concentration, while Darby et al. (2005) reported an increase of 8.4%. Although these incremental risk increases are statistically significant at the population level, they necessitate large cohorts for detection. In contrast, our study did not yield a significant association, aligning with findings from other regional studies, such as those conducted in Poland (Grzywa-Celińska et al., 2022) and China (Kudo et al., 2021). This lack of significant association can primarily be attributed to two pivotal factors: the indoor radon levels observed in the study population and the relatively small sample size. Previous research indicates a heightened risk of lung cancer among individuals exposed to indoor radon levels exceeding 200 Bq/m^3^ (Lorenzo-González et al., 2019; Torres-Durán et al., 2014), a threshold considerably greater than the average levels encountered in our study. Additionally, our study’s limited sample size—a common limitation in singular residential epidemiological studies—likely restricted the statistical power necessary to identify a significant association for a relatively infrequent outcome such as lung cancer (Keith et al., 2012). These findings underscore that while there exists robust evidence supporting a causal link between radon exposure and lung cancer, regional disparities in radon concentrations, variations in study design, and distinct population characteristics can considerably influence observed associations.

The study found a strong link between living close to gold mine tailings and an increased risk of lung cancer. People who lived further away from the gold mine tailings were less likely to develop lung cancer. These results support previous research from Spain, which also showed higher lung cancer risk among people living near mining sites (Fernández-Navarro et al., 2012). This association may be due to the release of carcinogenic pollutants like uranium from gold mine tailings into the air. These pollutants tend to have a limited dispersion, often impacting nearby populations (Chanda-Kapata, 2020). Despite the well-established causal link between smoking (both active and passive) and lung cancer risk, our study did not reveal a statistically significant association. This unexpected finding contradicts numerous prior studies that have consistently identified smoking as the primary risk factor for lung cancer (Mezzoiuso et al., 2021; Auvinen et al., 1996 71.Walser et al., 2008; Park et al., 2019; Nhu Ngoc et al., 2022). Most of these studies have reported a synergistic association between radon exposure and smoking, suggesting that individuals exposed to both factors may be at an elevated risk of developing lung cancer (Nhu Ngoc et al., 2022; Park et al., 2019). Potential explanations for this discrepancy could include the small sample size, limitations in smoking exposure assessment, or the possible presence of other confounding factors that were not adequately controlled. The overall prevalence of exposure to carcinogens, irritants, and toxins was relatively low in both groups, but individuals in the exposed group reported significantly higher rates of occupational exposure to these substances. These findings were consistent with previous research demonstrating limited occupational exposure to lung cancer-causing agents among participants (Auvinen et al., 1996; Torres-Durán et al., 2014). In the adjusted analysis, occupational exposure to carcinogens, irritants, and toxins emerged as a substantial risk factor for lung cancer, aligning with a Finnish study that found exposure to carcinogens was associated with a slight increase in the risk of lung cancer (Auvinen et al., 1996). This result suggested that occupational exposure play a crucial role in exacerbating the health risks associated with radon exposure.

### Chronic obstructive pulmonary diseases (COPD) and leukemia

Previous research has consistently demonstrated a strong correlation between indoor radon exposure and an elevated risk of developing certain types of cancer, particularly lung cancer. However, the association between residential radon exposure and other health conditions is still under active investigation. This study investigated the potential correlation between indoor radon exposure and two specific health conditions: leukemia and chronic obstructive pulmonary disease (COPD). This study also examined the relationship between different risk factors, including residential location, smoking, occupational exposure, and the incidence of Chronic Obstructive Pulmonary Disease (COPD) and leukemia among individuals living near gold mine tailings. While the initial analysis indicated a possible correlation between living near gold mine tailings and a higher risk of COPD, this association was not confirmed after considering the influence of other risk factors, such as smoking. The possible association between proximity to gold mine tailings and increased risk of respiratory diseases such as COPD was observed in previous studies conducted in the same settings as the present study (Iyaloo et al., 2020; Nkosi et al., 2015a, 2015b). Increased COPD risk in these areas could be attributed to various factors, including the release of fine particles from the tailings (The Bench Marks Foundation 2016; International Human Rights Clinic, 2016). These particles possess high airborne transport and floating capacity, enabling them to contaminate the air of the mining region and spread over vast distances (Da Silva-Rêgo et al., 2022).

Our findings revealed a significant association between indoor radon exposure and the development of leukemia. Individuals residing in homes with elevated radon levels were associated with increased leukemia risk compared to those living in homes with lower radon concentrations. These results corroborate previous studies indicating that radon exposure can induce genetic damage, a potential mechanism underlying the development of leukemia. Specifically, Zlobina et al. (2022), Oancea et al. (2017), and Schwartz and Klug (2016) have provided compelling evidence supporting the association between radon exposure and leukemia. In contrast, our analysis did not reveal a statistically significant association between indoor radon exposure and chronic obstructive pulmonary disease (COPD). Although prior studies in the United States have suggested a possible link (Turner et al., 2012; Wang et al., 2022; Yitshak-Sade et al., 2019), our findings, along with those of Spanish researchers (Barbosa-Lorenzo et al., 2017; Pando-Sandoval et al., 2022; Ruano-Ravina et al., 2021), indicated that the evidence for such a connection remains inconclusive. It’s crucial to note that the absence of a significant association in our study does not definitively rule out the possibility of a relationship between radon exposure and COPD. Further research are needed to address this issue, taking into account factors previously identified as potential confounders in the association between radon exposure and lung cancer.

Numerous literature has demonstrated a conclusive association between cigarette smoking and an increased risk of chronic obstructive pulmonary disease (COPD) and leukemia (Chung et al., 2023; Fircanis et al., 2014; Laniado-Laborín, 2008). However, the present study failed to find a statistically significant correlation between these variables. This contrasts with existing research that has consistently associated smoking with a higher likelihood of developing COPD (Chung et al., 2023; Fircanis et al., 2014). While our results may appear contradictory, they do not discount the well-established evidence of tobacco smoke’s detrimental effects on respiratory and hematological health (World Health Organization (WHO) 2009). It’s possible that our study lacked the power to detect a significant association, or that other factors, such as genetic predisposition or environmental exposures, may have affected the outcomes. Nevertheless, our findings emphasize the importance of future research to understand the complex relationship between smoking, genetics, and disease risk. Despite specific statistical results, the overwhelming consensus remains that reducing tobacco smoke exposure is a crucial public health measure to alleviate the burden of COPD and leukemia (Jha, 2020).

### Strengths and weaknesses of the study

The study employed a well-designed cross-sectional epidemiological approach to investigate the association between indoor radon exposure from gold mine tailings and health problems. It utilized calibrated radon monitors and structured questionnaires to enhance data accuracy and employed appropriate statistical methods to control for potential confounders. However, the cross-sectional design limited the ability to establish temporal relationships between exposure and health outcomes, highlighting the need for longitudinal studies to provide stronger evidence of causality. The reliance on self-reported data for health outcomes and other variables may have introduced measurement bias, potentially affecting the accuracy of the findings. Additionally, the study’s sample size, although reasonably large, may have been insufficient to detect small or moderate associations between exposure and health outcomes, especially for rare diseases. Using a larger sample size would have increased the statistical power of the study. Furthermore, relying on a single radon measurement per dwelling may not have accurately captured the variability of indoor radon levels over time. Multiple radon measurements would have provided a more reliable assessment of exposure. Finally, the study’s findings, focused on a specific community exposed to gold mine tailings, may not be directly applicable to other populations with different exposure sources or living conditions, necessitating further research to assess the generalizability of the results.

## Conclusion and recommendations

The research provides strong evidence of the health risks associated with indoor radon exposure in a community near gold mine tailings. Although the study showed significantly higher radon levels in the exposed group compared to the control group, there was no statistically significant association between radon exposure and lung cancer risk. However, the research did identify significant correlations between radon exposure and leukemia, as well as other risk factors such as residential location, smoking, and occupational exposure. These findings underscore the importance of implementing radon mitigation strategies, raising public awareness, and conducting further research to protect public health and minimize the adverse effects of radon exposure. By employing a comprehensive approach involving radon testing, remediation, policy development, and public education, we can effectively mitigate the health risks associated with this environmental hazard.

## Data Availability

All data are available upon request by emailing the corresponding author.
